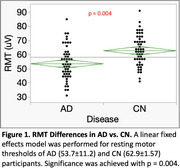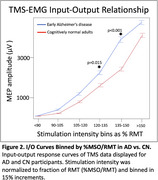# Input‐output curve reveals slope of cortical hyperexcitability in early Alzheimer’s disease

**DOI:** 10.1002/alz70862_110085

**Published:** 2025-12-23

**Authors:** Julia H. Cho, Brice Passera, Recep Ozdemir, Martina Upton, Emma Ferry, Daniel Z. Press, Mouhsin Shafi, Peter J. Fried, Stephanie S. Buss

**Affiliations:** ^1^ Harvard Medical School, Boston, MA USA; ^2^ Berenson‐Allen Center for Noninvasive Brain Stimulation, Beth Israel Deaconess Medical Center and Harvard Medical School, Boston, MA USA

## Abstract

**Background:**

Alzheimer’s disease (AD) is linked to abnormal cortical excitability. Recent studies have suggested that amyloid‐related cortical hyperexcitability may drive faster clinical decline and be related to spreading of tau. However, we lack non‐invasive methods to quickly and directly assay cortical excitability in AD patients. We generate an Input‐Output response curve (I/O Curve) using transcranial magnetic stimulation (TMS) with electromyography (EMG) to investigate mechanisms of cortical excitability in AD.

**Methods:**

Participants included 52 biomarker‐positive early AD (CDR=0.5‐1, age 70±8, 37% female) and 51 cognitively normal older adults (CN; age 70±6.5, 53% female). Single‐pulse TMS was applied to left motor cortex to measure resting motor threshold (RMT). An I/O Curve was generated by delivering 10 pulses each at eight stimulation intensities (30‐100% maximum stimulator output (%MSO)). We recorded motor‐evoked potentials (MEP) from the right first dorsal interosseous muscle. RMTs were compared between AD and CN using a linear model controlling for scalp‐to‐cortex distance (SCD) and protocol (i.e., EEG cap). For the I/O curve, stimulation intensity was normalized to RMT (%MSO/RMT) and binned in 15% increments. MEP amplitudes along the I/O Curve were analyzed using a linear mixed effects model with effects of Group, %MSO/RMT, and Group*%MSO/RMT; covariates of age, sex, education, and APOE4 alleles; and participant‐level random effects. Contrasts were conducted comparing the groups at each point along the I/O curve.

**Results:**

RMT was lower in AD than in CN (η² *p* =0.12, medium effect size, *p* = 0.004, Figure 1); SCD was a significant covariate (*p* = 0.006). The I/O Curve revealed an increased response in the AD group, with significant effects of Group (η² *p* =0.10, medium effect size; *p* = 0.007), %MSO/RMT (η² *p* =0.71, *p* <0.001), and Group*%MSO/RMT (η² *p* =0.05, small effect size, *p* = 0.013). Covariates were not significant. Increased excitability in AD was greatest at higher stimulation intensities (120‐135%MSO/RMT: Cohen’s d=0.286, small effect size, *p* = 0.015; 135‐150%MSO/RMT: Cohen’s d=0.495, medium effect size, *p* <0.001, Figure 2).

**Conclusions:**

TMS‐EMG confirms evidence of increased cortical excitability in AD. This was evident at lower intensities with the RMT model (implicating voltage‐gated Na+ channels) and higher intensities with the I/O Curve (implicating AMPA receptors). TMS may be useful to measure target engagement of novel therapies targeting cortical excitability in AD.